# Efficacy and safety of decitabine in treatment of elderly patients with acute myeloid leukemia: A systematic review and meta-analysis

**DOI:** 10.18632/oncotarget.17241

**Published:** 2017-04-19

**Authors:** Pin-Fang He, Jing-Dong Zhou, Dong-Ming Yao, Ji-Chun Ma, Xiang-Mei Wen, Zhi-Hui Zhang, Xin-Yue Lian, Zi-Jun Xu, Jun Qian, Jiang Lin

**Affiliations:** ^1^ Laboratory Center, Affiliated People's Hospital of Jiangsu University, Zhenjiang 212002, Jiangsu, P.R. China; ^2^ The Key Lab of Precision Diagnosis and Treatment of Zhenjiang City, Zhenjiang 212002, Jiangsu, P.R. China; ^3^ Department of Clinical Laboratory, Affiliated People's Hospital of Jiangsu University, Zhenjiang 212002, Jiangsu, P.R. China; ^4^ Department of Hematology, Affiliated People's Hospital of Jiangsu University, Zhenjiang 212002, Jiangsu, P.R. China

**Keywords:** acute myeloid leukemia (AML), decitabine, elderly patient, systematic review, meta-analysis

## Abstract

Elderly patients with acute myeloid leukemia (AML) have limited treatment options concerned about their overall fitness and potential treatment related mortality. Although a number of clinical trials demonstrated benefits of decitabine treatment in elderly AML patients, the results remains controversial. A meta-analysis was performed to evaluate efficacy and safety of decitabine in treatment of elderly AML patients. Eligible studies were identified from PubMed, Web of Science, Embase and Cochrane Library. Nine published studies were included in the meta-analysis, enrolling 718 elderly AML patients. The efficacy outcomes were complete remission (CR), overall response rate (ORR) and overall survival (OS). Safety was evaluated based on treatment related grades 3–4 adverse events (AEs) and early death (ED) rate. Pooled estimates with 95% confidence interval (CI) for CR, ORR and OS were 27% (95% CI 19%–36%), 37% (95% CI 28%–47%) and 8.09 months (95% CI 5.77–10.41), respectively. The estimated treatment related early death (ED) incidences were within 30-days 7% (95% CI 2%–11%) and 60-days 17% (95% CI 11%–22%), respectively. Thrombocytopenia was the most common grades 3–4 AEs. Subgroup analyses of age, cytogenetics risk, AML type and bone marrow blast percentage showed no significant differences of treatment response to decitabine. In conclusion, decitabine is an effective and well-tolerated therapeutic alternative with acceptable side effects in elderly AML patients.

## INTRODUCTION

Acute myeloid leukemia (AML) is a heterogeneous malignant disease, characterized by clonal abnormal proliferation of hematopoietic stem or progenitor cells, causing marrow failure and death within months or even weeks if diagnosis and treatment time is delayed [1]. The incidence of AML increases progressively with age, and advanced age is an adverse prognostic factor in AML patients [2, 3]. Poor prognosis of elderly AML patients is due to several different factors, including comorbid conditions, decreased organ function, poor performance status and a higher incidence of adverse karyotypes [4, 5]. Although intensive chemotherapy can bring a high rate of complete remission (CR) in elderly AML patients, the probable toxicity and fatal side effects limit the extensive application in elderly patients unfit for intensive therapy [6].

Decitabine (5-aza-2′-deoxycytidine, dacogen), a hypomethylating agent, is a deoxynucleoside analogue of cytidine which has hypomethylating effect on DNA at low-dose. Hypomethylating agents, demethylating the promoters of tumor suppressor genes and reactivating their expression, have shown potential roles in the treatment of newly diagnosed myeloid malignancies [7]. Decitabine is firstly approved for the treatment of patients with Myelodysplastic syndrome (MDS) and is also introduced for the treatment of unfit AML patients. Decitabine is already approved in the European Union for AML patients aged > 65 years who are not eligible for standard induction chemotherapy. Whereas it is not approved in the United States to treat this same group of patients, since a multicenter phase III study was failed statistically to show that elderly AML patients received decitabine lived longer than control group. Multiple clinical trials have been taken to evaluate the treatment advantages of decitabine in elderly AML patients. The purpose of this study was to assess what is currently known about the efficacy and safety of decitabine in elderly AML patients by performing a meta-analysis.

## MATERIALS AND METHODS

### Evidence identification

We performed a comprehensive search of relevant published studies from databases including PubMed, Web of Science, Embase and Cochrane Library. Additional literatures were further investigated through manual search from relevant reference lists to identify any relevant trials. The search strategy was based on the following combined MeSH terms: (“decitabine” or “5-aza-2′-deoxycytidine” or “dacogen”) and (“acute myeloid leukemia” or “AML”) and (“older” or “elderly”). Restrictions were made on publication language (English only) and population (AML patients aged over 60 years). The publication year was not restricted, and final update of the search was conducted on February 2017.

### Study selection and data extraction

Publications were eligible for this meta-analysis if they met the following inclusion criteria: (1) original research investigated decitabine in treatment of elderly AML; (2) elderly patients aged ≥ 60 years and met the diagnostic criteria for AML; (3) decitabine used as monotherapy in previously untreated elderly AML patients; (4) provided sufficient information on at least one of the following survival outcomes: CR, overall response rate (ORR) and overall survival (OS); (5) studies are clinical trial, and at least 10 or more patients included in the trial. To minimize bias in the selected literatures, two reviewers independently checked each full-text paper for eligibility according to the same inclusion criteria. Any disagreements were further discussed and resolved by consensus or third party arbitration. General information extracted from eligible studies including first author's name, year of publication, country of origin, trial design, and exact number of patients. The efficacy endpoints of interest were survival outcomes, including CR, ORR and OS. The safety endpoints were treatment related grades 3–4 adverse events (AEs) and early death (ED) rate.

### Definition of outcomes and risk of bias in individual studies

Outcomes were defined according to the 2003 revised International Working Group response criteria [8]. CR was defined by the presence of the following: with < 5% blasts in bone marrow, with > 1.0 × 10^9^/L neutrophils and > 100 × 10^9^ /L platelet in peripheral blood, and without evidence of extramedullary leukemia. ORR was composed of complete remission, complete remission with incomplete leukocyte or platelet recovery (CRi) and partial response. OS was measured from the date of entry onto a study until death from any cause or was censored at the last follow-up. Each included study was individually assessed risk of bias according to The Cochrane Collaboration's Risk of Bias Assessment Tool following characteristics: sequence generation, allocation concealment, blinding of participants, incomplete outcome data, selective outcome reporting, other sources of bias [9].

### Statistical analysis

The presence of heterogeneity was quantified using the *I*^2^ statistic, which calculates values between 0 and 100%. Higher value indicates a greater degree of heterogeneity. As a cut-off *P* > 0.05 or *I*^2^ < 50%, fixed-effects model was applied. Otherwise, random-effects model was applied. Estimated proportions (ES) with 95% confidence intervals (CIs) were calculated for ratio outcomes. Dichotomous variables were pooled by odds ratio (OR) as an effective measurement. Considering some significant prognosis factors, subgroup analyses for response rate were performed based on age, cytogenetics risk, AML type and bone marrow blast if relevant data were available. Funnel plots were inappropriate to present as the total number of included studies were 9 (< 10). Sensitivity analyses were performed by deletion of each single study to evaluate stability of the results. We conducted all the statistical analyses by using Stata software, version 12.0 (Stata Corp, College Station, TX, USA).

## RESULTS

### Study characteristics

**Figure 1:**
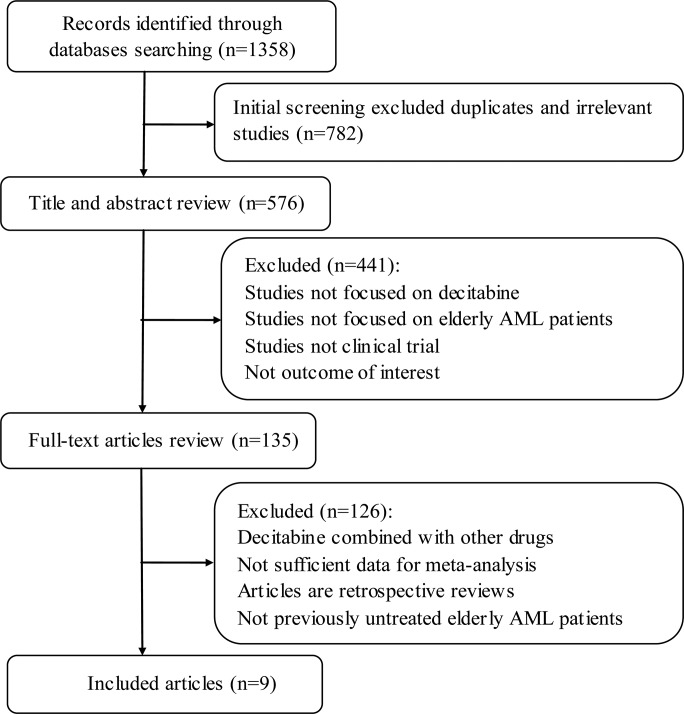
Flow diagram of the included studies selection AML, Acute myeloid leukemia.

A total of 1,358 citations were identified using the initial search strategy, and detailed selection process was illustrated in Figure 1. The yielded 9 studies including 718 patients ultimately qualified for our inclusion criteria in this meta-analysis [10–18]. Characteristics of included studies were summarized in Table 1. Publication years of these studies were ranged from 2010 to 2016. Decitabine treatment schedule varied in the 9 trials. Outcomes of efficacy and safety endpoints in the included patients were presented in Table 2. Baseline characteristics of eligible patients including age, gender, AML type, cytogenetics risk and bone marrow blast were described in Supplementary Table 1.

**Table 1:** General characteristics of the included studies

First Author	Year	Country	Study-center	Phase	Dose and schedule of decitabine	Trial Sponsor
Jacob et al. [10]	2015	India	NR	NR	20 mg/m^2^ 5-days 4 weeks	NR
Yan et al. [11]	2012	America	Single-center	Phase II	20 mg/m^2^ 10-days 4 weeks	National Cancer Institute
Ritchie et al. [12]	2013	America	Single-center	NR	20 mg/m^2^ 10-days 4 weeks	Leukemia Fighters™
Cashen et al. [13]	2010	America	Multicenter	Phase II	20 mg/m^2^ 5-days 4 weeks	NR
Blum et al. [14]	2010	America	Single-center	Phase II	20 mg/m^2^ 10-days 4 weeks	National Cancer Institute
Tawfik et al. [15]	2014	America	Single-center	NR	20 mg/m^2^ 5-days 4 weeks	National Cancer Institute
Kantarjian et al. [16]	2012	America	Multicenter	Phase III	20 mg/m^2^ 5-days 4 weeks	MDACC and others
Lübbert et al. [17]	2011	Germany	Multicenter	Phase II	15 mg/m^2^ 3-days 6weeks*	European LeukemiaNet
Park et al. [18]	2016	Korea	Single-center	NR	20 mg/m^2^ 5-days 4 weeks	Yonsei University

Abbreviations: NR: Not Reached; 15 mg/m^2^ 3-days 6weeks*: 15 mg/m^2^, three times daily on 3 consecutive days. MDACC: M.D. Anderson Cancer Center.

**Table 2:** Outcomes of efficacy and safety endpoints in the included patients

First Author	No. patients	Median age (years)	Gender (male%)	CR rate	ORR	OS (month) range	ED rate	
							30-days	60days
Jacob et al. [10]	15	65	80	NA	NA	5.5 (1.5–13)	0.067	0.067
Yan et al. [11]	16	75	50	0.563	0.563	NA	NA	NA
Ritchie et al. [12]	52	75	44	0.404	0.404	10.3(8.8–11.6)	0.058	0.154
Cashen et al. [13]	55	74	51	0.240	0.250	7.7(5.7–11.6)	NA	NA
Blum et al. [14]	53	74	64	0.470	0.640	13.7(9–18)	0.02	0.15
Tawfik et al. [15]	34	75	50	0.180	0.265	3.4(1.3–7.4)	0.15	0.38
Kantarjian et al. [16]	242	73	57	0.157	0.277	7.7(6.2–9.2)	0.09	0.197
Lübbert et al. [17]	227	72	61	0.132	0.260	NA	NA	0.128
Park et al. [18]	24	73	50	0.250	0.500	NA	NA	NA

Abbreviations: No.: Number; NA: Not Available; CR: complete remission; ORR: overall response rate; OS: overall survival; ED: early death.

### Efficacy

**Figure 2:**
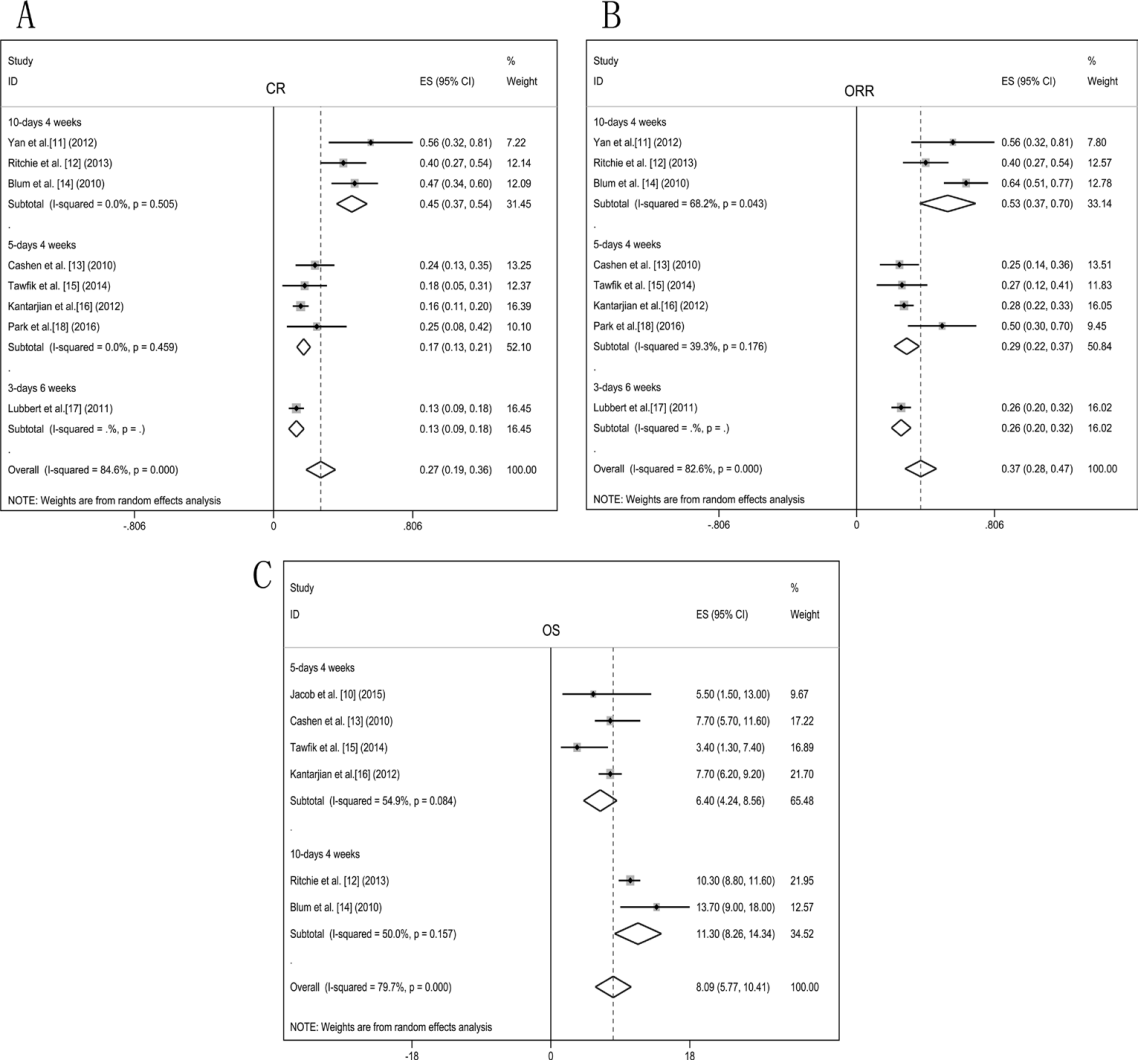
Forest plots of efficacy endpoints of the decitabine treated elderly AML patients Pooled estimated proportions (95% confidence interval) were generated with random effects models and the respective forest plots are reported: (**A**) complete remission (CR); (**B**) overall response rate (ORR); (**C**) overall survival (OS).

Eligible studies were pooled into one dataset for meta-analysis, random-effects models were used to calculate response rates and survival. CR rates were assessed in eight articles [11–18]. Pooled estimate for overall CR rate was 27% (95% CI 19%–36%, Figure 2A). In subgroup analysis of therapy schedule, data from 3-days 6 weeks course showed that CR rate was 13% (95% CI 9%–18%), and the 5-days 4 weeks course showed a CR rate of 17% (95% CI 13%–21%). The patients treated with 10-days 4 weeks course achieved a significantly higher CR rate of 45% (95% CI 37%–54%) than the other two courses (*P* < 0.001).

ORR was evaluated in eight studies [11–18]. Pooled estimate for ORR of decitabine treated patients was 37% (95% CI 28%–47%, Figure 2B). Subgroup analysis of ORR with 3-days 6 weeks course was 26% (95% CI 20%–32%) and 5-days 4 weeks course was 29% (95% CI 22%–37%). Patients treated with the 10-days 4 weeks course showed a relatively higher ORR of 53% (95% CI 37%–70%). In the different treatment schedule, ORR presented a consistent pattern with CR, 10-days 4 weeks course showed significantly better response than the other two courses (*P* = 0.001).

The OS of elderly AML patients treated with decitabine was analysed in six articles [10, 12–16]. Pooled estimate of OS was 8.09 months (95% CI 5.77–10.41, Figure 2C). In subgroup analysis of therapy schedule, OS of 5-days 4 weeks course was 6.40 months (95% CI 4.24–8.56) and 10-days 4 weeks course was 11.30 months (95% CI 8.26–14.34). Subgroup analysis showed that 10-days 4 weeks course achieved a relatively prolonged survival.

### Safety

**Figure 3:**
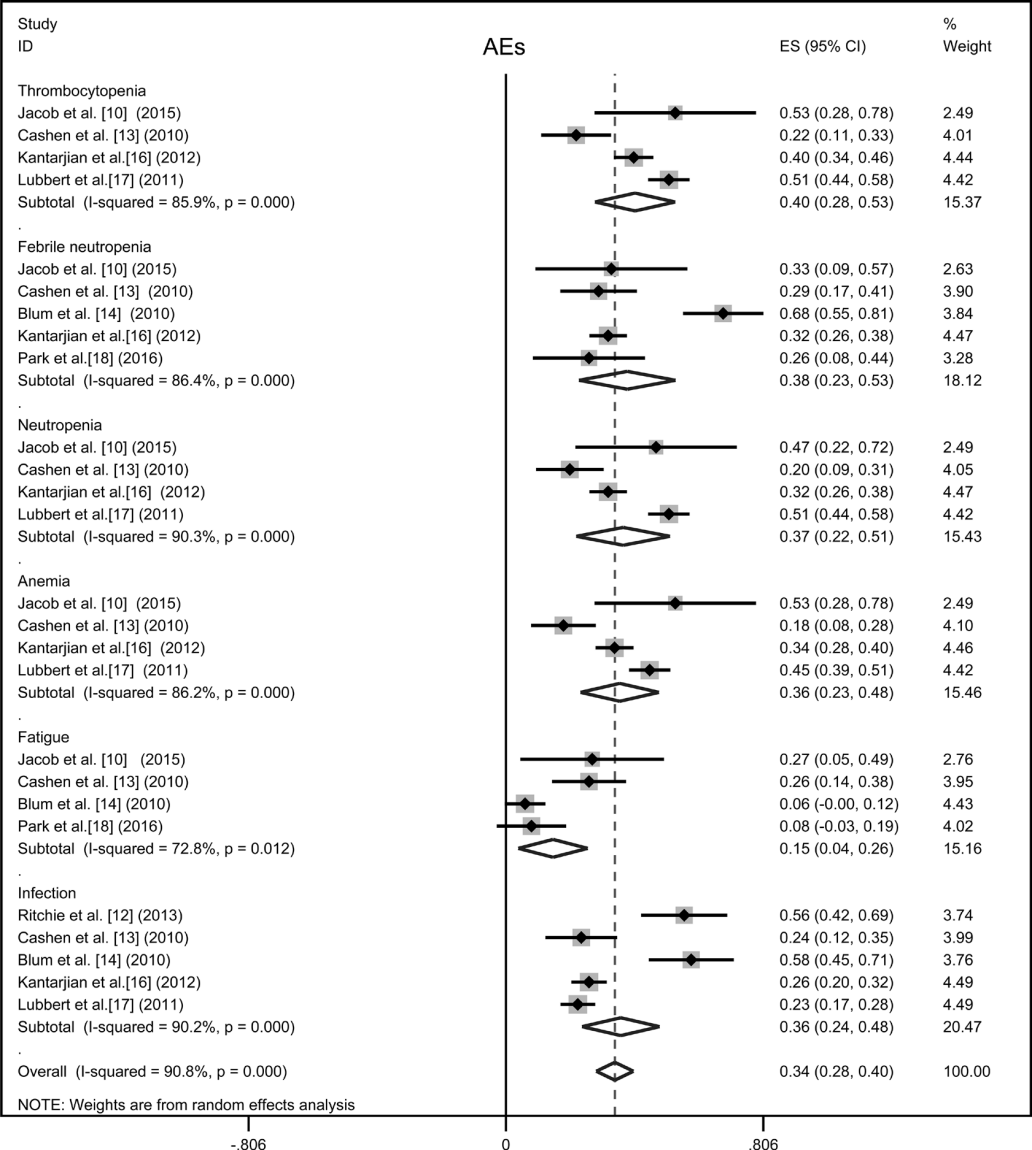
Forest plot of the estimated proportions of most frequent grade 3-4 adverse events (AEs) Pooled estimated proportions (95% confidence interval) were generated with random effects model.

Regarding toxicity of decitabine, seven studies appraised treatment related grades 3–4 AEs [10, 12–14, 16–18], random-effects model was applied. Myelosuppression was the most common toxicity observed in decitabine treated patients. The reported high risks of treatment related AEs were presented in Figure 3: thrombocytopenia 40% (95% CI 28%–53%), febrile neutropenia 38% (95% CI 23%–53%), neutropenia 37% (95% CI 22%–51%), anemia 36% (95% CI 23%–48%) and fatigue 15% (95% CI 4%–26%). Occurrence of treatment associated infections was 36% (95% CI 24%–48%), pneumonia (25%) and sepsis (9%) were the most frequent infectious complications.

**Figure 4:**
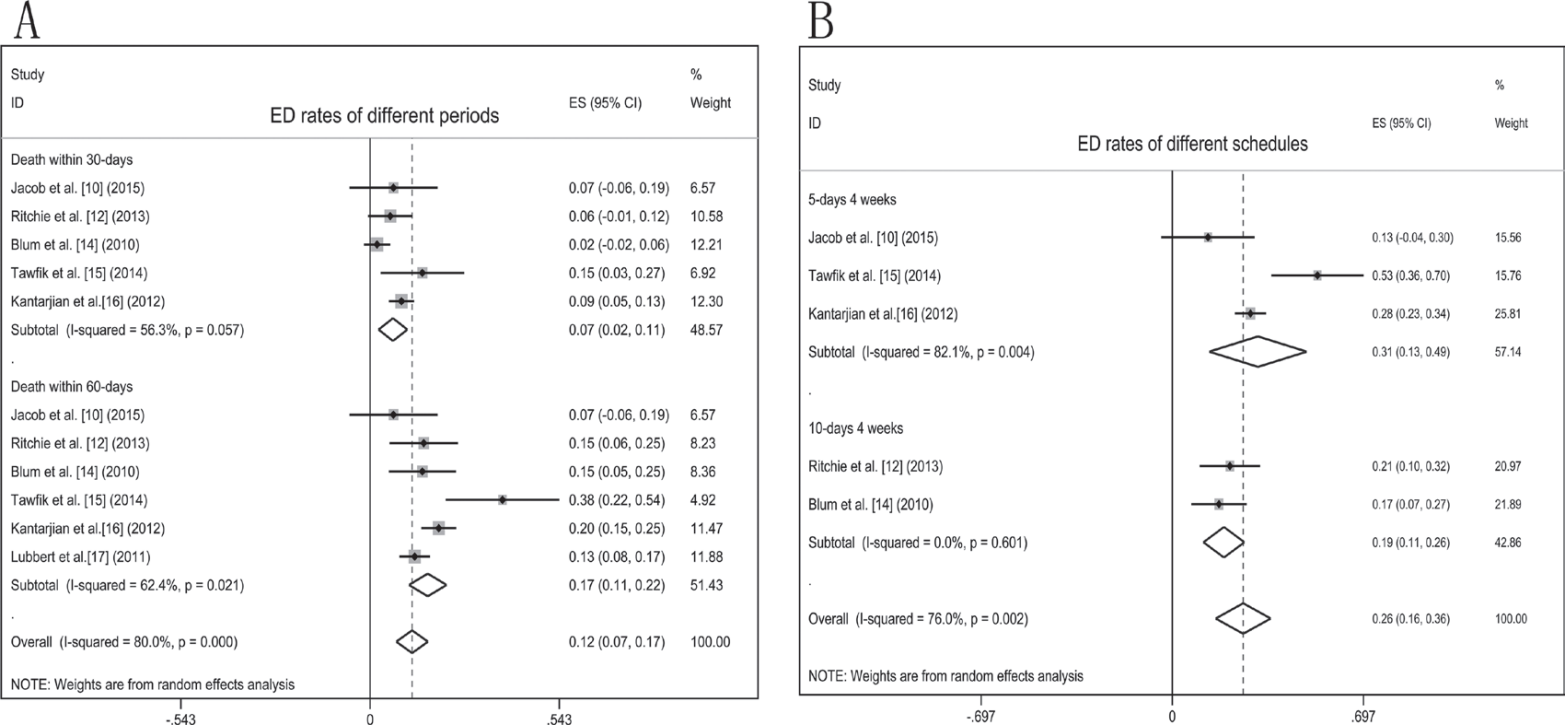
Forest plots of ED rates in elderly AML patients treated with decitabine Pooled estimated proportions (95% confidence interval) were generated with random effects models: (**A**) ED rates of different periods; (**B**) ED rates of different decitabine schedules.

Decitabine treatment related ED rates were analysed in six studies [10, 12, 14–17], random-effects model were adopted. Death within 30-days was 7% (95% CI 2%–11%) and 60-days mortality was 17% (95% CI 11%–22%, Figure 4A). Subgroup analysis of the association between ED rate and decitabine course with 5-days and 10-days was 31% (95% CI 13%-49%) and 19% (95% CI 11%–26%), respectively (Figure 4B). ED rates analyses showed that there was no significant difference in mortality between 5-days and 10-days courses treatment (*P* = 0.072).

### Subgroup analyses

**Figure 5:**
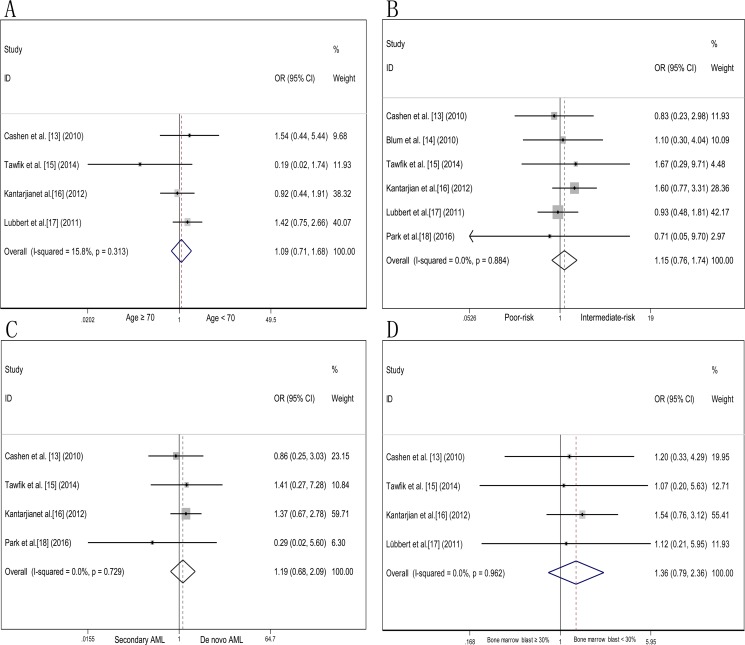
Odds ratio of decitabine response in AML patients according to age, cytogenetics risk, AML type and bone marrow blast percentage (**A**) Odds ratio of decitabine treatment response in elderly AML patients aged < 70 years and ≥ 70 years. (**B**) Odds ratio of decitabine treatment response in elderly AML patients cytogenetically profiled with intermediate-risk and poor-risk. (**C**) Odds ratio of decitabine treatment response in elderly patients diagnosed with de novo AML and secondary AML. (**D**) Odds ratio of decitabine treatment response in elderly AML patients with bone marrow blast < 30% and bone marrow blast ≥ 30%.

Subgroup analyses of age, cytogenetics risk, AML type and bone marrow blast percentage of response to decitabine were performed (Figure 5). A slightly higher proportion of patients with more advanced age (≥ 70 years), odds ratio of patients aged < 70 versus those aged ≥ 70 was 1.09 (95% CI 0.71–1.68, *P* = 0.691), the two age groups showed equivalent effect to decitabine. Sub-analysis of cytogenetics risk, response odds ratio of intermediate-risk versus poor-risk was 1.15 (95% CI 0.76–1.74, *P* = 0.497), favorable-risk was not available to evaluate because of insufficient data. Treatment response of patients diagnosed with de novo AML compared secondary AML, odds ratio was 1.19 (95% CI 0.68–2.09, *P* = 0.552), suggesting that decitabine have similar effects to the two AML type. Odds ratio of patients with bone marrow blast < 30% and bone marrow blast ≥ 30% was 1.36 (95% CI 0.97–2.36, *P* = 0.266), decitabine treatment response did not show significant difference in patient with discrepant bone marrow blast percentage.

### Risk of bias within studies and sensitivity analyses

Based on the risk of bias assessment criteria, included 9 studies were classified into class B. Detailed assessment information was summarized in Supplementary Table 2. Sensitivity analyses indicated that excluding any single study did not significantly affect the pooled outcomes, suggesting the results of our meta-analysis were stable.

## DISCUSSION

Elderly AML patients are generally less capable of tolerating intensive cytotoxic induction and post-remission chemotherapy. The development of hypomethylating agents provides an alternative treatment strategy [19–28]. This meta-analysis showed that decitabine brought considerable treatment response in elderly AML patients. Preliminary data indicated longer exposure times to decitabine showed an improved response rate and relatively prolonged survival. The dose schedule of decitabine did not seem to affect ED rate with patients receiving 10-days decitabine (19%) compared with those received 5-days course (31%). Neutropenia and thrombocytopenia related to myelosuppression were common during decitabine treatment. Prospective clinical trials that directly compared decitabine courses are still needed to confirm the more optimal administration.

Age and cytogenetics risk are important prognostic impact factors of AML patients, this meta-analysis suggests that decitabine can overcome the negative prognostic factors such as advanced age, unfavorable cytogenetic features and even bone marrow blasts ≥ 30%. In addition, chemotherapy outcomes for secondary AML are generally dismal, but decitabine can present comparable effect of de novo AML and secondary AML. Another important factor affecting the prognosis of patients is gene mutations, controversy still exists on predictive value of genetic characteristics (DNA methylation changes and mutations in DNMT3A, TET2, IDH1, IDH2, ASXL1 and expression of miR-29b) to guide decitabine therapy in previous studies [11, 14, 29–33]. Recently, Welch reported that all patients with TP53 mutation had a response to 10-day courses of decitabine, and 67% of patients with unfavorable-risk cytogenetic responded to decitabine treatment, survival rate among patients with unfavorable-risk were similar to those with intermediate-risk [34]. TP53 mutations tend to be occurred in older patients with AML, whereas TP53 mutations are usually associated with poor prognosis and low response to standard cytotoxic therapy [35–36]. Welch's trial suggested that decitabine could induce favourable clinical response in patients with AML who have TP53 mutations and who have unfavorable cytogenetic profile.

Decitabine demonstrated activity alone and with combination in elderly AML patients [37–45]. A multicenter, randomized phase III trial indicated that decitabine achieved a higher response rate and a trend toward improved OS compared with low-dose cytarabine or supportive care (7.7 months vs 5.0 months, respectively. *P* = 0.108) in AML patients aged ≥ 65 years [16]. Decitabine also showed considerable effect compared with intensive chemotherapy in two retrospective studies [21–22]. Gupta N et al and Quintás CA et al reported that intensive chemotherapy could lead higher response rates but no statistically significant differences in OS compared with decitabine induction in older AML patients, and treatment-related ED rate was not statistically different in the two groups. These studies again suggest the potential role of decitabine in elderly patients with AML who cannot tolerate intensive chemotherapy. A phase II, open label trial from China reported an encouraging result in elderly AML patients treated with decitabine in combination with cytarabine, aclarubicin, and granulocyte-colony stimulating factor (decitabine-CAG) [45]. Decitabine-CAG combination regimen showed favorable response rates (CR 64.7% and ORR 82.4%) and low treatment-related adverse effects (induction mortality 4.4%). A significantly longer median OS in patients with response (16 months) was observed. This study suggests that development of more effective strategies is imperative.

Limitations of our analysis should be considered when interpreting the outcomes. First, despite the same inclusion criteria, significant heterogeneity was detected. Second, sample size of subgroup analyses was relatively small. Third, we didn't explore gene mutation and treatment response interactions because of the insufficient data. Finally, considering a higher quality and credibility of eligible studies, only published studies were included. Confounding cannot be avoided and should be considered in this meta-analysis. Despite these limitations, our review is the first comprehensive meta-analysis of all eligible studies on the efficacy and safety of decitabine treated elderly AML patients.

In conclusion, our meta-analysis suggests that decitabine is an effective and well-tolerated therapeutic alternative with acceptable side effects in elderly AML patients. To improve the overall response and maintain durable remission, further studies should focus on determining the best administration schedule and developing the optimal combination with decitabine.

## SUPPLEMENTARY MATERIALS TABLES



## Abbreviations

AML: acute myeloid leukemia
95% CI: confidence interval
CR: complete remission
ORR: overall response rate
OS: overall survival
ED: early death
AEs: adverse events
OR: odds ratio
