# Supplementary material for: Efficacy and safety of decitabine in treatment of elderly patients with acute myeloid leukemia: A systematic review and meta-analysis
Source: Oncotarget. 2017 Apr 19;8(25):41498–507. doi: 10.18632/oncotarget.17241 (PMC5522197; doi:10.18632/oncotarget.17241)
Supplement: Supplementary file 1 [file oncotarget-08-41498-s001.pdf]

Efficacy and safety of decitabine in treatment of elderly patients with acute myeloid leukemia: A systematic review and meta-analysis

Supplementary Materials

Supplementary Table 1: Baseline characteristics of patients in the included studies

First Author	No. patients	Median age (years)	Gender (male %)	AML type (%)		BM blast (%)		Cytogenetics-risk (%)		
				De novo	secondary	< 30	≥ 30	favorable	intermediate	poor
Jacob et al. [10]	15	65	80	87	13	13	60	33	47	20
Yan et al. [11]	16	75	50	NR	NR	31	69	NR	NR	NR
Ritchie et al. [12]	52	75	44	NR	NR	NR	NR	NR	53	45
Cashen et al. [13]	55	74	51	23	71	33	67	NR	65	35
Blum et al. [14]	53	74	64	NR	NR	NR	NR	40	NR	30
Tawfik et al. [15]	34	75	50	53	41	56	35	3	32	38
Kantarjian et al. [16]	242	73	57	64	36	27	71	NR	63	36
Lübbert et al. [17]	227	72	61	49	51	3	95	1	45	32
Park et al. [18]	24	73	50	92	8	NR	NR	13	67	13

BM blast: Bone Marrow blast; NR: Not Reported;

Supplementary Table 2: Risk of bias assessment results

Study	Random sequence generation	Allocation concealment	Blinding of participants and personnel	Complete outcome data	No selective reporting	Other bias
Jacob et al. [10]	−	−	−	−	+	−
Yan et al. [11]	−	−	−	−	+	−
Ritchie et al. [12]	−	−	+	+	+	−
Cashen et al. [13]	+	+	+	+	+	−
Blum et al. [14]	−	−	−	+	+	−
Tawfik et al. [15]	−	+	+	+	+	−
Kantarjian et al. [16]	+	+	+	+	+	−
Lübbert et al. [17]	+	+	+	−	+	−
Park et al. [18]	−	−	−	−	+	−

Based on the risk of bias assessment criteria, each article was divided into three different categories:
A: the articles that meet all the criteria are classified into class A;
B: if more than one criterion was not reached or was unclear, we ranked it as class B;
C: for article does not reach any of the criteria, we divided it into class C.
Risk of bias assessment of the 9 studies showed that all the included studies were classified as B.
